# Social Media Sharing of Articles About Measles in a European Context: Text Analysis Study

**DOI:** 10.2196/30150

**Published:** 2021-11-08

**Authors:** Dominik Wawrzuta, Mariusz Jaworski, Joanna Gotlib, Mariusz Panczyk

**Affiliations:** 1 Department of Education and Research in Health Sciences Medical University of Warsaw Warsaw Poland

**Keywords:** measles, Facebook, Twitter, Pinterest, social media, vaccine, infodemiology, public health

## Abstract

**Background:**

Despite the existence of an effective vaccine, measles still threatens the health and lives of many Europeans. Notably, during the COVID-19 pandemic, measles vaccine uptake declined; as a result, after the pandemic, European countries will have to increase vaccination rates to restore the extent of vaccination coverage among the population. Because information obtained from social media are one of the main causes of vaccine hesitancy, knowledge of the nature of information pertaining to measles that is shared on social media may help create educational campaigns.

**Objective:**

In this study, we aim to define the characteristics of European news about measles shared on social media platforms (ie, Facebook, Twitter, and Pinterest) from 2017 to 2019.

**Methods:**

We downloaded and translated (into English) 10,305 articles on measles published in European Union countries. Using latent Dirichlet allocation, we identified main topics and estimated the sentiments expressed in these articles. Furthermore, we used linear regression to determine factors related to the number of times a given article was shared on social media.

**Results:**

We found that, in most European social media posts, measles is only discussed in the context of local European events. Articles containing educational information and describing world outbreaks appeared less frequently. The most common emotions identified from the study’s news data set were fear and trust. Yet, it was found that readers were more likely to share information on educational topics and the situation in Germany, Ukraine, Italy, and Samoa. A high amount of anger, joy, and sadness expressed within the text was also associated with a higher number of shares.

**Conclusions:**

We identified which features of news articles were related to increased social media shares. We found that social media users prefer sharing educational news to sharing informational news. Appropriate emotional content can also increase the willingness of social media users to share an article. Effective media content that promotes measles vaccinations should contain educational or scientific information, as well as specific emotions (such as anger, joy, or sadness). Articles with this type of content may offer the best chance of disseminating vital messages to a broad social media audience.

## Introduction

### Background

The first measles vaccine was approved in 1963. Before the invention of this vaccine, measles caused 6 million deaths annually [1]. Since use of this vaccine became common, the number of measles cases worldwide has started to decline. A few years after the first vaccination campaign, the number of new measles cases plunged to one-twentieth of the previous figure [2]. In some countries, the cases of measles have been eliminated almost completely, although local epidemics still occur from time to time [3].

Despite the proven effectiveness of vaccination in saving millions of lives annually, some individuals have questioned the safety and long-term benefits of vaccine use. In 1998, Andrew Wakefield, a British physician and academic, published an article connecting the measles, mumps, and rubella (MMR) vaccine to autism among children [4]. This paper was retracted 12 years later; however, antivaccine activists still argue against vaccination. Antivaccination movements are one of the main obstacles for public health professionals in conducting vaccination campaigns. Vaccine hesitancy is a significant problem as only a high measles vaccination coverage—of about 95%—can enable the complete eradication of this disease. Currently, global vaccination coverage against measles is approximately 70%. Measles vaccines protect not only human lives but also the economies of low- and middle-income countries, generating US $58 for an investment of US $1 [5].

One example of the result of the decline in measles vaccination coverage was the outbreak in Samoa. In September 2019, in Samoa, a country with a small population of 200,000, a measles outbreak led to over 5000 infections and 83 deaths [6]. The reason behind the outbreak was the suspension of the country's immunization program by the Samoan government in July 2018 following the death of 2 children as a result of nurses’ inadvertent use of curare muscle relaxant anesthetic instead of water to dilute the MMR vaccine. This led to a decrease in measles vaccination coverage in Samoa to 31% by the end of 2019 [7]. After multiple measles-related deaths, the authorities decided to organize a vaccination campaign. Approximately 95% of eligible people in Samoa were vaccinated against measles, which put an end to the outbreak [8]. This case shows how quickly the measles virus can spread when a vaccination program is suspended. Therefore, it is important to constantly monitor measles epidemiology and people’s attitudes toward it to promptly prevent vaccine hesitancy.

This problem surrounding vaccination coverage has also been observed in Europe. The European Centre for Disease Prevention and Control (ECDC) data suggests that the second dose of MMR vaccine coverage is over 95% in only 5 European Union countries. This extent of coverage can ensure the immunity of the population against this disease and eliminate the chance of an outbreak [9]. In the European Union, the number of measles cases declined in 2020—mainly caused by the COVID-19 pandemic and the result of wearing masks, practicing social distancing, and conducting social lockdowns [10]—except for Romania and Bulgaria. In 2020, there were over 20 measles cases per million inhabitants in these 2 countries [11]. After the COVID-19 pandemic ends, European societies will probably notice an increase in measles morbidity as there was a decline in measles vaccine administration among children during the lockdowns [12]. Moreover, Europeans have been using social media more frequently during the COVID-19 pandemic. This may result in the growth of negative attitudes in the public toward vaccination as exposure to disinformation on social media increases vaccine hesitancy [13,14]. As social media contribute significantly, analyzing social media content and the activities of users can help better understand public attitudes and opinions regarding measles. The knowledge gathered from this analysis will inform the actions to increase MMR vaccine coverage and prevent the spread of misinformation.

### Study Aim

We aim to characterize European measles news reports shared on social media platforms (ie, Facebook, Twitter, and Pinterest) during 2017 to 2019 (ie, the pre–COVID-19 period). For this purpose, we formulated the following 3 research questions: (1) What are the main topics of the articles on measles published in the European Union countries? (2) What sentiments are associated with these news articles? (3) Which features of the articles are associated with an increased number of shares on social media?

## Methods

### Data Collection and Preparation

We collected articles on measles that were shared on Facebook, Twitter, or Pinterest from 28 European Union countries.

First, we translated the word “measles” into all 23 official languages of the European Union using Wiktionary [15] and used a social media data analysis platform (ahrefs, Ahrefs Pte. Ltd [16]), which continuously collects, processes, and stores information from social media platforms about users’ content and activities, to collect news articles. For each European Union country, we downloaded all articles on measles published from January 1, 2017, to December 31, 2019. We chose this time range because there was a significant increase in the number of measles infections in Europe in 2017 that lasted until the start of the COVID-19 pandemic [17]. We selected articles containing the word “measles” in the national language and published on websites with national domains (eg, “.de” domain for Germany, “.pl” for Poland). Our data set contained the URL of the article, the publication date, and the number of shares (ie, the total number of shares for all instances of the article) on Facebook, Twitter, and Pinterest.

As the next step, we obtained the full text of the articles (n=12,638) and read the content. To accomplish this task, we used a Python newspaper package (version 0.3.0) [18] that allows an automated download of the website content.

We automatically translated all non-English articles into English using Yandex Translate [19]. Finally, we removed all duplicate articles and those that had been improperly downloaded or translated. Our final data set comprised 10,305 articles. This dataset, containing the text of translated news, country of origin, and the total number of shares, was published and publicly made available on the Zenodo platform [20]. Finally, we processed the data in order to be able to apply the latent Dirichlet allocation (LDA) method. We used R packages (tidytext [21] and textstem [22]) to tokenize text; remove numbers, punctuation, and English stop words; and lemmatize all words.

### Statistical Analysis

#### Topic Modeling

We used LDA [23] to identify the main topics of the 10,305 articles in our data set. We trained multiple LDA models with a different number of topics (ranging from 1 to 40). We then analyzed perplexity and coherence levels to select the model that best describes our data set. In the next step, 2 researchers individually labeled the topics chosen by LDA to categorize them. The researchers analyzed not only the keywords assigned to each topic but also the content of the 20 articles with the highest amount of contribution to the topic. Initially, they independently described each topic with a freely chosen category. Then, they analyzed the created categories (without knowing the topic they were assigned to) and together created a unified set of categories (eg, education, Europe, and the world). Finally, they classified the topics again with a new set of categories. In this final stage, there were no discrepancies in assessment.

#### Sentiment Analysis

We calculated the main emotions associated with each article using the syuzhet R package [24]. This package uses the National Research Council Canada (NRC) Word-Emotion Association lexicon. The NRC lexicon is a set of 14,182 English words that are not just concerned with polarity (reporting positive or negative words) but associated with 8 fundamental emotions introduced by Plutchik [25] (ie, anger, anticipation, disgust, fear, joy, sadness, surprise, and trust) [26]. These words were labeled manually by crowdsourcing—each word could be associated with more than one emotion). The sentiment of an article is the sum of emotions related to the words that make it up. The occurrence of each word from one of the categories in the article translates as “1” in the sentiment score for that category. Finally, each article is scored for each sentiment category [27].

#### Linear Regression

We considered a linear regression model to find the factors determining the number of shares of an article on social media (ie, the dependent variable). We used forward selection regression to create our model. We also used Cook distance method to identify and remove outliers [28] and variance inflation factor to check the existence of collinearity [29]. As independent variables, we used topics generated by the LDA model, the emotions related to the articles, the number of new national measles cases in the month when an article was published, the population of the country, and the percentage of active social media users in each country. The number of new monthly measles cases for each country was collected from the ECDC website [30]. Data on the populations of European countries were obtained from the Eurostat database [31], and the proportion data of active social media users in each country were acquired from the Statista website [32].

## Results

### Sample Description

After article selection and data processing, we had a final sample of 10,305 measles-related articles, published between January 1, 2017, and December 31, 2019, in European Union countries. The highest number of published articles retrieved was from Italy, but the articles that were the most shared ones were from the United Kingdom. Table 1 shows the number of articles from each country, and the sum and average number of shares. In Table S1 of Multimedia Appendix 1, we present more detailed yearly aggregated data.

**Table 1:** Description of collected data (N=10,305).

Country	Articles, n (%)	Shares	
		Total	Mean per article (SD)
Austria	200 (1.94)	34,539	173 (425)
Belgium	250 (2.43)	89,442	358 (1095)
Bulgaria	404 (3.92)	23,418	58 (346)
Croatia	53 (0.51)	8395	158 (281)
Republic of Cyprus	9 (0.09)	250	28 (52)
Czech Republic	51 (0.49)	78,043	1530 (3154)
Denmark	114 (1.11)	92,603	812 (2274)
Estonia	13 (0.13)	2546	196 (349)
Finland	119 (1.15)	86,076	723 (2136)
France	1252 (12.15)	633,111	506 (1826)
Germany	1132 (10.98)	914,665	808 (4420)
Greece	697 (6.76)	57,082	82 (792)
Hungary	116 (1.13)	28,851	249 (568)
Ireland	167 (1.62)	32,226	193 (389)
Italy	2025 (19.65)	1,462,172	722 (2983)
Latvia	3 (0.03)	927	309 (520)
Lithuania	1 (0.01)	156	156 (0)
Luxembourg	20 (0.19)	2609	130 (212)
Malta	0 (0)	0	N/A^a^
Netherlands	313 (3.04)	68,705	220 (607)
Poland	216 (2.10)	46,658	216 (1042)
Portugal	964 (9.35)	200,812	208 (1116)
Romania	544 (5.28)	44,363	82 (459)
Slovakia	21 (0.20)	4300	205 (255)
Slovenia	25 (0.24)	2450	98 (232)
Spain	664 (6.44)	954,613	1438 (18,634)
Sweden	253 (2.46)	86,958	344 (1361)
United Kingdom	679 (6.59)	2,012,118	2963 (23,726)

^a^N/A: not applicable.

### Topic Modeling

We found that 13 is the best number of topics to describe all collected news articles, accounting for the perplexity and coherence values (Table S2 of Multimedia Appendix 1). After labeling, we discovered that these topics can be aggregated into 3 clusters. The first group contains the topics featured in educational articles, which describe the signs and symptoms of measles infection, debunk antivaccine claims, and explain scientific advancements in the prevention of this disease. The topics in the second group are related to European information, which contain country-specific information on measles outbreaks and health policies. The last group includes topics related to countries outside Europe, including measles cases in the United States, and the Samoa measles outbreak. Table 2 presents the main words connected with specific topics and their classification to general groups.

**Table 2:** Topics, their classification, and key words.

Topic	Main words	Group classification
Topic 1	immune, immune_system, study, cell, system, researcher, cancer, antibody, virus, memory	Education
Topic 2	child, measles, hospital, Samoa, epidemic, Sweden, people, disease, campaign, patient	World
Topic 3	county, York, measles, city, orthodox, Jewish, disease, USA, Brooklyn, confirm	World
Topic 4	measles, rash, symptom, fever, infection, disease, day, infect, cough, virus	Education
Topic 5	vaccine, parent, autism, child, ani, polio, Wakefield, diphtheria, study, vaccinate_child	Education
Topic 6	MMR, GP, December, England, measles, UK, dose_MMR, jab, HSE, NHS	Europe
Topic 7	vaccination, school, federal, Spahn, measles_vaccination, Germany, day_care, measles, CDU, mandatory	Europe
Topic 8	DGS, health, confirm, directorate, measles, outbreak, age, Portugal, UK, Lisbon	Europe
Topic 9	hospital, measles, Italy, health, Catania, ship, vaccinate, Sicily, hospitalize, region	Europe
Topic 10	country, world, Europe, organization, world_health, health_organization, European, measles, increase, Ukraine	Europe
Topic 11	measles, Roma, CDC, health, dose, Greece, vaccine, population, diseases, Spain	Europe
Topic 12	France, measles, health, agency, Aquitaine, epidemic, health_France, vaccinate, Poitiers, people	Europe
Topic 13	obligation, dolphin, Cicciobello, decree, sport, doll, Burioni, certification, time, market	Europe

**Figure 1:**
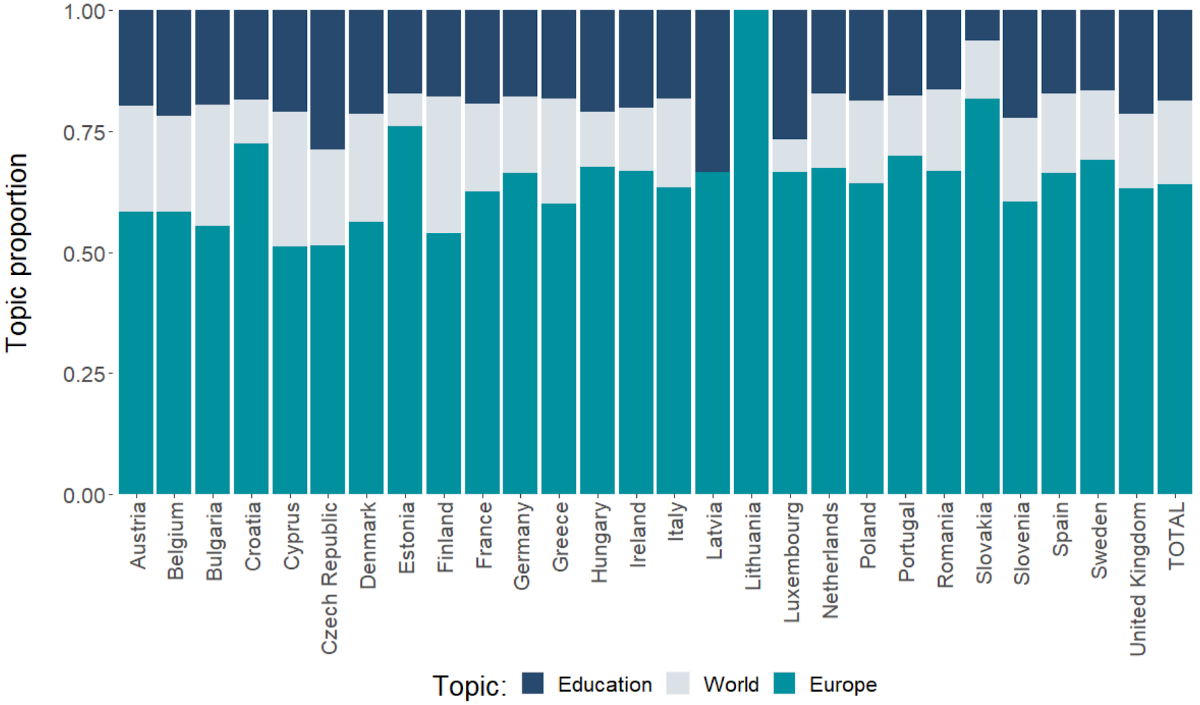
Distribution of topics across all countries analzyed.

Using these 3 clustered meta-topics, we evaluated the popularity of all topics in each country. Figure 1 shows that all of the European countries mainly write about the topic in the European context. Educational topics are more popular than world topics, although the difference between the two varies between countries. In Multimedia Appendix 1, we present sample articles that were highly connected to specific topics.

### Sentiment Analysis

**Figure 2:**
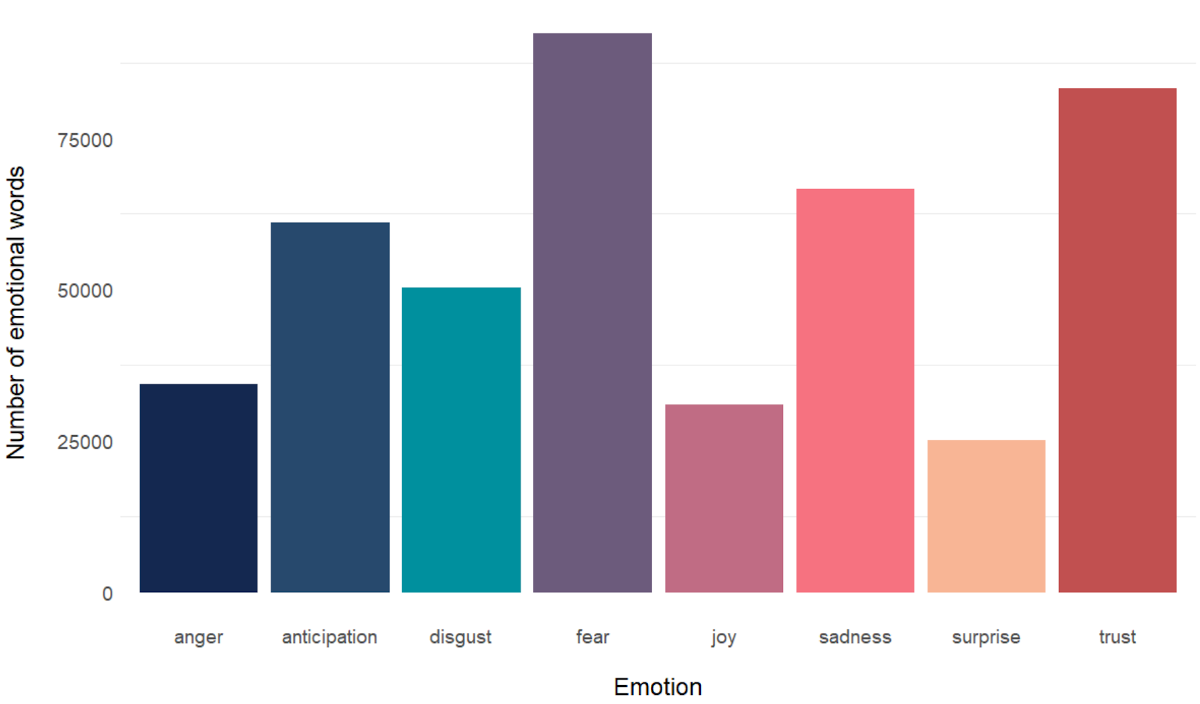
Number of words with a certain sentiment in the data set of articles analyzed.

We analyzed what emotions are connected to measles-related articles in Europe. Figure 2 shows the frequency of appearance of the words associated with a specific emotion in our data set. The most common emotions were fear and trust.

We also determined which words contributed the most to the emotion levels in our data set. For each emotion, we reviewed the 15 most popular words from our data set, as shown in Table 3.

**Table 3:** Emotions and connected words.

Emotion	Connected words
Fear	disease, infection, death, hospital, epidemic, risk, infectious, contagious, die, medical, fever, prevent, bear, warn, emergency
Trust	accord, hospital, school, doctor, medical, immunization, county, system, organization, authority, recommend, ministry, continue, level, director
Sadness	disease, death, hospital, epidemic, infectious, die, late, sick, emergency, illness, leave, mother, fatal, fall, bad
anticipation	child, death, time, patient, epidemic, risk, public, medical, organization, result, continue, warn, start, prevention, develop
Disgust	disease, death, epidemic, infect, infectious, contagious, rash, cough, sick, bad, ill, nose, elimination, lose, treat
Anger	disease, death, epidemic, bear, fatal, eradicate, bad, ill, force, fear, elimination, victim, lose, treat, fight
Joy	child, organization, ministry, baby, majority, infant, mother, childhood, achieve, safe, grow, treat, create, save, progress
Surprise	death, epidemic, organization, warn, emergency, infant, leave, alarm, expect, catch, lose, treat, sneeze, break, vote

Evidently, fear is connected to words describing the harmfulness of measles. Trust is associated with the words related to health care systems and protection. *Sadness* is conveyed when describing the adverse effects of the disease; *anticipation* is conveyed when talking about the actions being taken to reduce the spread of the disease; *disgust* is associated with the characteristics and signs of measles; *anger* is about the fight against measles; *joy* is about children and protecting them from the disease; and *surprise* is about emergent events. In Multimedia Appendix 1, we present sample articles highly connected to specific emotions.

### Linear Regression

We used linear regression to identify the variables related to the number of shares of an article on social media. Table 4 presents the results of the forward selection regression. In the multicollinearity analysis, all variance inflation factor scores were determined to be lower than 5. Therefore, we assumed that there is no collinearity among variables. Detailed results are presented in Multimedia Appendix 1.

We discovered that readers were more likely to share information on topics 1, 4, and 5, which convey educational information. Topics 2, 7, 10, and 13 describe the situation in Samoa, Germany, Ukraine, and Italy, and these topics were also positively associated with the number of total shares. Topic 6, which describes the situation in the United Kingdom, resulted in a reduced number of shares. Topic 4, which describes the signs and symptoms of measles, had the highest impact on the average of shareability.

With regard to emotions, on average, a higher proportion of anger, joy, and sadness expressed in an article was associated with a higher number of article shares, whereas surprise in the article was associated with reduced number of shares. Moreover, articles published in countries with a higher population and a higher number of active social media users understandably received more shares on social media.

**Table 4:** Regression results (adjusted R^2^=0.04852).

Variable	Beta	Standard error	*t* value (*df*)^a^	*P* value
Constant	–1994	282	–7.08	<.001
Topic 1	1025	218	4.695	<.001
Topic 2	711	127	5.615	<.001
Topic 4	1833	145	12.651	<.001
Topic 5	475	196	2.426	.015
Topic 6	–512	171	–2.994	.003
Topic 7	1024	122	8.42	<.001
Topic 10	616	114	5.388	<.001
Topic 12	184	124	1.487	.14
Topic 13	375	181	2.072	.04
Anger	54	15	3.512	<.001
Joy	53	14	3.66	<.001
Surprise	–50	20	–2.551	.01
Sadness	38	13	2.828	.005
Disgust	–24	15	–1.603	.11
Social media users	22	4	5.112	<.001
Population	0.000018	0.000001	9.393	<.001

^a^*df*=10,254.

## Discussion

### Sample Description

The number of articles published from 2017 to 2019 varies across the different countries included in this study. The highest number of articles was published in Italy, which is likely because of the high number of measles cases reported in Italy. During this period, a total of 9252 measles cases were reported in Italy [30]—the highest reported in the European Union. The articles published in the United Kingdom received the greatest number of shares, which might be attributed to the popularity of the English language worldwide. In our data set, less than one relevant article per month was published in Malta, Slovenia, Slovakia, Lithuania, Estonia, Latvia, and Luxembourg. These countries have small populations, and most reported a low number of measles cases during 2017 to 2019. Latvia had a relatively higher number of measles cases, but as was observed (Table 1), despite the low number of measles-related articles published, they were extensively shared on social media. Topic Modeling

European media mostly published news about local events, reporting on almost all significant outbreaks of measles in Europe. However, several of the events received special media attention. The decision of the German government to make the measles vaccine mandatory in response to an increase in measles cases has been frequently discussed in those articles [33]. The measles outbreak in the Aquitaine region in France, caused by insufficient vaccination coverage, is also one of the most described events in the media [34]. Furthermore, measles clusters in the United Kingdom, Portugal, Italy, and Greece are also frequently mentioned in these articles. Some of these articles describe the case of the Cicciobello doll in Italy. The Cicciobello doll is a toy that pretends to be suffering from measles and that children can cure with plasters or cream. Experts have criticized this doll for banalizing such a severe disease [35].

The theme of world news is dominated by 2 events: One is the measles outbreak in the Orthodox Jewish community in New York [36], and the second is the measles outbreak in Samoa [7]. Both these events are interesting because they are concerned with relatively small outbreaks. During the years of analysis, millions of people contracted measles in Africa, Asia, and South America, but this did not attract the attention of European media. As indicated by our data set, media attention was mainly drawn to outbreaks in small, specific communities, and not necessarily to events that had the greatest impact on the lives of millions of people.

Educational themes focus mainly on 3 threads. Some articles describe the symptoms of measles, reflecting the readers’ interest in the signs, symptoms, and causes of the disease and their desire to recognize them. The second topic is related to the scientific findings on measles, including studies on the potential oncolytic activity of this virus [37]. The last educational topic dispels the doubts—raised by Wakefield’s paper, which has since been retracted—related to the risk of autism allegedly caused by the MMR vaccine [4]. Moreover, another paper published in 2019, of a nationwide cohort study conducted in Denmark that found that MMR vaccination does not increase the risk for autism, was widely discussed in the media [38].

### Sentiment Analysis

In the course of human life, up to 34,000 different emotions can be distinguished [39]. Psychologist Robert Plutchik proposed the theory of 8 basic emotions, which have developed evolutionarily and are innate in humans and help them survive [25,40]. As a result of combining these basic emotions, more complex emotions responsible for specific experiences arise (eg, joy + trust = love; trust + fear = submission). Basic emotions are triggered by specific events, eliciting an appropriate response that has an evolutionary function. Table 5 presents the characteristics of these basic emotions as proposed by Plutchik [40].

**Table 5:** Characteristics of basic emotions.

Emotion	Stimulus event	Behavioral reaction	Function	Opposite emotion
Joy	Gain of a valued object	Retain or repeat	Gain resources	Sadness
Trust	Member of the group	Groom	Mutual support	Disgust
Fear	Threat	Escape	Safety	Anger
Surprise	Unexpected event	Stop	Gain time to orient	Anticipation
Sadness	Loss of a valued object	Cry	Reattach to the lost object	Joy
Disgust	Unpalatable object	Vomit	Eject poison	Trust
Anger	Obstacle	Attack	Destroy obstacle	Fear
Anticipation	New territory	Map	Knowledge of territory	Surprise

Emotions are an important part of media articles, as emotional stories attract readers’ attention [41]. The most common emotion identified from all collected articles was *fear*. The creation of fear-based articles is consistent with the results of studies that show that fear-related news reports automatically attract public attention [42]. In the case of the articles analyzed in this study, the emotion *fear* was mainly concerned with the fear of the disease and its consequences. This emotion is so strong that sometimes the fear of disease is worse for a patient than the disease itself [43].

The analyzed articles were also highly connected with *trust*. There is a natural link between fear and trust because it is in response to concerns about a dangerous disease that we place our trust in the health care system and the vaccines that protect us against the pathogen. Research conducted during the Ebola epidemic showed that trust in the health care system increased during the outbreak [44].

In the field of marketing, the relationship between the basic emotions in the text and its potential to go viral has often been examined. An analysis of 7000 articles published in The New York Times revealed that positive content tends to go viral more than negative content. More specifically, this study has demonstrated that sad content is less likely to go viral, whereas articles expressing anger or anxiety result in higher number of shares on social media [45]. Teixeira [46] analyzed thousands of reactions to several advertisements and found that maintaining viewers’ engagement levels is associated with the emotions of joy and surprise. Libert and Tynski [47] also found that emotional activation is the key to viral success. They used Plutchik’s set of emotions [25] and found that negative emotions are not commonly present in highly viral content. Surprise and anticipation are also extremely common in highly viral content.

### Linear Regression

We identified a few features of news articles that are associated with an increased number of shares on social media. All educational topics are positively connected with the number of shares. This shows that social media users generally prefer to share general educational news over informational ones. This finding is consistent with the results of previous research, wherein it was proved that Facebook users are more likely to share “soft” news related to children, health, and education than “hard” news related to politics or urgent occurrences [48]. Sharing educational and scientific articles can also be associated with the willingness to increase one’s credibility, self-confidence, and self-esteem from other social media users [49].

The results also show that information on the events in Sweden, Germany, Italy, and Ukraine was shared frequently, whereas the publication of information on the situation in the United Kingdom was negatively associated with the number of social media shares. The interest in measles in Ukraine may have been generated as a result of the country having the highest number of measles cases in Europe. In 2017-2019, the number of measles cases in Ukraine was around 100,000 [50]. This situation was similar to the interest in measles cases in Italy, where the highest number of cases among all countries in the European Union was reported during the study period [30]. Furthermore, research conducted by Facebook showed that country-based or cultural differences have an impact on Facebook activity. In the United Kingdom, social media users are younger but less active than those in Germany or Sweden [51].

The findings of previous research analyzing what type of web-based content become viral are generally in line with our results. They indicate that articles containing positive emotions or anger are more likely to be shared [45].

### Conclusions

Articles on measles shared on social media in Europe primarily report on European events, and only a small proportion of articles report on educational news or international measles-related events. The international events mainly describe outbreaks that have occurred in a small number of infected people but are interesting from an epidemiological point of view. The distribution of topics covered by the media is similar across all European Union countries.

In this study, the two main emotions expressed in the analyzed measles-related articles were fear and trust. These emotions appeared in the articles most frequently. However, these emotions were not associated with frequent sharing of articles on social media. We found that an article has a high probability to drive public discourse if it contains educational or scientific information, as well as specific emotions (ie, anger, joy, or sadness). Making media content based on these principles can facilitate the creation of effective messages against measles vaccine hesitancy. Articles that follow these principles offer the best chance of disseminating information to a broad audience on social media and influencing the mindset of the public regarding vaccines.

## Appendix

Multimedia Appendix 1 Supplementary tables providing details of the analyses conducted.

## Abbreviations

ECDC: The European Centre for Disease Prevention and Control
LDA: latent Dirichlet allocation
MMR: measles, mumps, and rubella

## References

[1] Goodson JL, Seward JF (2015). Measles 50 years after use of measles vaccine. Infect Dis Clin North Am.

[2] Conis E (2019). Measles and the modern history of vaccination. Public Health Rep.

[3] Patel MK, Dumolard L, Nedelec Y, Sodha SV, Steulet C, Gacic-Dobo M, Kretsinger K, McFarland J, Rota PA, Goodson JL (2019). Progress toward regional measles elimination - Worldwide, 2000-2018. MMWR Morb Mortal Wkly Rep.

[4] Godlee F, Smith J, Marcovitch H (2011). Wakefield's article linking MMR vaccine and autism was fraudulent. BMJ.

[5] Durrheim DN (2020). Measles eradication—retreating is not an option. Lancet Infect Dis.

[6] Craig AT, Heywood AE, Worth H (2020). Measles epidemic in Samoa and other Pacific islands. The Lancet Infectious Diseases.

[7] Isaacs D (2020). Lessons from the tragic measles outbreak in Samoa. J Paediatr Child Health.

[8] WHO (2019). Measles Outbreak in The Pacific - Situation Report No. 9. Joint WHO/UNICEF Measles Outbreak Response and Preparedness in the Pacific.

[9] Vaccination coverage for the second dose of measles-containing vaccine, EU/EEA, 2018. European Centre for Disease Prevention and Control.

[10] Wu D, Liu Q, Wu T, Wang D, Lu J (2020). The impact of COVID-19 control measures on the morbidity of varicella, herpes zoster, rubella and measles in Guangzhou, China. Immun Inflamm Dis.

[11] Measles notification rate per million population by country, January 2020 - December 2020. European Centre for Disease Prevention and Control.

[12] Santoli JM, Lindley MC, DeSilva MB, Kharbanda EO, Daley MF, Galloway L, Gee J, Glover M, Herring B, Kang Y, Lucas P, Noblit C, Tropper J, Vogt T, Weintraub E (2020). Effects of the COVID-19 pandemic on routine pediatric vaccine ordering and administration - United States, 2020. MMWR Morb Mortal Wkly Rep.

[13] Wilson SL, Wiysonge C (2020). Social media and vaccine hesitancy. BMJ Glob Health.

[14] Wawrzuta D, Jaworski M, Gotlib J, Panczyk M (2021). Characteristics of antivaccine messages on social media: systematic review. J Med Internet Res.

[15] Wiktionary.

[16] Ahrefs - SEO Tools & Resources to Grow Your Search Traffic.

[17] Number of cases of measles reported monthly in the European Economic Area (EEA) from 1999 to 2021. Statista.

[18] Ou-Yang L News, full-text, and article metadata extraction in Python 3. GitHub.

[19] Yandex Translate. yandex.com.

[20] Wawrzuta D European articles about measles. Dataset on Zenodo.

[21] Silge J, Robinson D (2016). tidytext: text mining and analysis using tidy data principles in R. JOSS.

[22] Rinker T Tools for fast text stemming & lemmatization.

[23] Blei D, Ng A, Jordan M (2003). Latent Dirichlet allocation. Journal of Machine Learning Research.

[24] Jockers M Introduction to the syuzhet package.

[25] Plutchik R (1980). A general psychoevolutionary theory of emotion. Emotion: Theory, research, and experience: Vol. 1. Theories of emotion.

[26] Mohammad SM, Turney PD NRC Emotion Lexicon.

[27] Naldi M (2019). A review of sentiment computation methods with R packages. arXiv.

[28] Stevens JP (1984). Outliers and influential data points in regression analysis. Psychol Bull.

[29] Thompson CG, Kim RS, Aloe AM, Becker BJ (2017). Extracting the variance inflation factor and other multicollinearity diagnostics from typical regression results. Basic Appl Soc Psych.

[30] Measles. European Centre for Disease Prevention and Control.

[31] Population on 1 January by age and sex. Eurostat.

[32] Tankovska H Active social media penetration in selected European countries in 2020. Statista.

[33] Connolly K German parliament approves compulsory measles vaccinations. The Guardian.

[34] Bernadou A, Astrugue C, Méchain M, Le Galliard V, Verdun-Esquer C, Dupuy F, Dina J, Aït-Belghiti F, Antona D, Vandentorren S (2018). Measles outbreak linked to insufficient vaccination coverage in Nouvelle-Aquitaine region, France, October 2017 to July 2018. Eurosurveillance.

[35] Monella L, Cuddy A Italy’s measles doll sparks controversy. Euronews.

[36] McDonald R, Ruppert P, Souto M, Johns D, McKay K, Bessette N, McNulty L, Crawford J, Bryant P, Mosquera M, Frontin S, Deluna-Evans T, Regenye D, Zaremski E, Landis V, Sullivan B, Rumpf B, Doherty J, Sen K, Adler E, DiFedele L, Ostrowski S, Compton C, Rausch-Phung E, Gelman I, Montana B, Blog D, Hutton B, Zucker H (2019). Notes from the field: measles outbreaks from imported cases in orthodox Jewish communities - New York and New Jersey, 2018-2019. MMWR Morb Mortal Wkly Rep.

[37] Russell L, Peng K (2018). The emerging role of oncolytic virus therapy against cancer. Chin Clin Oncol.

[38] Hviid A, Hansen JV, Frisch M, Melbye M (2019). Measles, mumps, rubella vaccination and autism. Ann Intern Med.

[39] (2020). Managing emotions. Deloitte.

[40] Plutchik R (2001). The Nature of Emotions: Human emotions have deep evolutionary roots, a fact that may explain their complexity and provide tools for clinical practice. Am Sci.

[41] Bagozzi RP, Gopinath M, Nyer PU (1999). The role of emotions in marketing. J Acad Mark Sci.

[42] Schmidt LJ, Belopolsky AV, Theeuwes J (2015). Potential threat attracts attention and interferes with voluntary saccades. Emotion.

[43] Singh T, Newby DE (2021). Is the fear of disease worse than the disease itself?. Heart.

[44] Nuriddin A, Jalloh M, Meyer E, Bunnell R, Bio F, Jalloh M, Sengeh P, Hageman K, Carroll D, Conteh L, Morgan O (2018). Trust, fear, stigma and disruptions: community perceptions and experiences during periods of low but ongoing transmission of Ebola virus disease in Sierra Leone, 2015. BMJ Global Health.

[45] Berger J, Milkman Kl (2012). What Makes Online Content Viral?. J Mark Res.

[46] Teixeira T (2012). The New Science of Viral Ads. Harvard Business Review.

[47] Libert K, Tynski K (2013). Research: The Emotions that Make Marketing Campaigns Go Viral. Harvard Business Review.

[48] Kalsnes B, Larsson AO (2017). Understanding news sharing across social media. Journalism Studies.

[49] Lee CS, Ma L, Goh DHL, Zhong N, Callaghan V, Ghorbani AA, Hu B (2011). Why do people share news in social media?. Lecture Notes in Computer Science.

[50] Rodyna R (2019). Measles situation in Ukraine during the period 2017-2019. European Journal of Public Health.

[51] Cheng J, Burke M, de Gant B (2021). Country Differences in Social Comparison on Social Media. Proc ACM Hum Comput Interact.

