# Supplementary material for: Social Media Sharing of Articles About Measles in a European Context: Text Analysis Study
Source: J Med Internet Res. 2021 Nov 8;23(11):e30150. doi: 10.2196/30150 (PMC8663483; doi:10.2196/30150)
Supplement: Multimedia Appendix 1 [file jmir_v23i11e30150_app1.doc]

Table S1
Number of articles, the total number of shares, proportions of topics, and most popular emotions in the countries each year

| **Country** | **Year** | **Number of articles** | **Sum of shares** | **Education topic** | **Europe topic** | **World topic** | **Most popular emotion** |
| --- | --- | --- | --- | --- | --- | --- | --- |
| **Austria** | 2017 | 11 | 1847 | 0.23 | 0.18 | 0.59 | trust |
|  | 2018 | 28 | 4820 | 0.17 | 0.27 | 0.56 | fear |
|  | 2019 | 161 | 27872 | 0.2 | 0.21 | 0.59 | fear |
| **Belgium** | 2017 | 26 | 9875 | 0.21 | 0.31 | 0.48 | fear |
|  | 2018 | 25 | 5044 | 0.21 | 0.04 | 0.75 | fear |
|  | 2019 | 199 | 74532 | 0.22 | 0.2 | 0.58 | fear |
| **Bulgaria** | 2017 | 102 | 1763 | 0.27 | 0.18 | 0.55 | fear |
|  | 2018 | 26 | 7005 | 0.13 | 0.25 | 0.62 | trust |
|  | 2019 | 276 | 14650 | 0.18 | 0.27 | 0.55 | fear |
| **Croatia** | 2017 | 5 | 1250 | 0.13 | 0.02 | 0.85 | fear |
|  | 2018 | 39 | 5280 | 0.19 | 0.08 | 0.73 | fear |
|  | 2019 | 9 | 1865 | 0.22 | 0.16 | 0.63 | trust |
| **Republic of Cyprus** | 2017 | 3 | 224 | 0.01 | 0.33 | 0.65 | anticipation |
|  | 2018 | 3 | 25 | 0.17 | 0.36 | 0.46 | fear & trust |
|  | 2019 | 3 | 1 | 0.44 | 0.13 | 0.42 | fear |
| **Czech Republic** | 2017 | 7 | 13824 | 0.07 | 0.17 | 0.76 | fear |
|  | 2018 | 13 | 16179 | 0.37 | 0.14 | 0.49 | fear |
|  | 2019 | 31 | 48040 | 0.31 | 0.23 | 0.47 | fear |
| **Denmark** | 2017 | 17 | 43403 | 0.21 | 0.37 | 0.42 | fear |
|  | 2018 | 19 | 5624 | 0.18 | 0.23 | 0.59 | fear |
|  | 2019 | 78 | 43576 | 0.22 | 0.19 | 0.59 | fear |
| **Estonia** | 2017 | 3 | 1410 | 0.05 | 0.28 | 0.67 | fear |
|  | 2018 | 6 | 85 | 0.26 | 0 | 0.74 | fear |
|  | 2019 | 4 | 1051 | 0.13 | 0 | 0.87 | fear |
| **Finland** | 2017 | 31 | 13642 | 0.12 | 0.27 | 0.62 | fear |
|  | 2018 | 52 | 63266 | 0.2 | 0.31 | 0.49 | fear |
|  | 2019 | 36 | 9168 | 0.2 | 0.25 | 0.55 | fear |
| **France** | 2017 | 112 | 50939 | 02. | 0.2 | 0.6 | fear |
|  | 2018 | 488 | 202980 | 0.19 | 0.19 | 0.62 | fear |
|  | 2019 | 652 | 379192 | 0.2 | 0.17 | 0.63 | fear |
| **Germany** | 2017 | 145 | 62762 | 0.19 | 0.12 | 0.69 | fear |
|  | 2018 | 141 | 32685 | 0.17 | 0.17 | 0.66 | fear |
|  | 2019 | 846 | 819218 | 0.18 | 0.16 | 0.66 | trust |
| **Greece** | 2017 | 292 | 21029 | 0.19 | 0.21 | 0.59 | fear |
|  | 2018 | 240 | 6141 | 0.18 | 0.24 | 0.58 | fear |
|  | 2019 | 165 | 29912 | 0.17 | 0.19 | 0.64 | fear |
| **Hungary** | 2017 | 38 | 8199 | 0.25 | 0.2 | 0.55 | trust |
|  | 2018 | 47 | 11536 | 0.17 | 0.08 | 0.74 | trust |
|  | 2019 | 31 | 9116 | 0.22 | 0.06 | 0.73 | trust |
| **Ireland** | 2017 | 20 | 2013 | 0.26 | 0.17 | 0.58 | fear |
|  | 2018 | 77 | 13022 | 0.18 | 0.14 | 0.68 | fear |
|  | 2019 | 70 | 17191 | 0.2 | 0.11 | 0.68 | fear |
| **Italy** | 2017 | 670 | 411532 | 0.17 | 0.18 | 0.65 | fear |
|  | 2018 | 761 | 663324 | 0.19 | 0.18 | 0.63 | fear |
|  | 2019 | 594 | 387316 | 0.19 | 0.19 | 0.63 | fear |
| **Latvia** | 2017 | 0 | 0 | 0 | 0 | 0 | - |
|  | 2018 | 1 | 7 | 0.02 | 0 | 0.98 | trust |
|  | 2019 | 1 | 920 | 0.49 | 0 | 0.5 | anticipation |
| **Lithuania** | 2017 | 0 | 0 | 0 | 0 | 0 | - |
|  | 2018 | 0 | 0 | 0 | 0 | 0 | - |
|  | 2019 | 1 | 156 | 0 | 0 | 1 | fear |
| **Luxembourg** | 2017 | 1 | 81 | 0 | 0 | 1 | fear |
|  | 2018 | 1 | 31 | 0.08 | 0 | 0.91 | trust |
|  | 2019 | 18 | 2497 | 0.29 | 0.08 | 0.63 | disgust |
| **Malta** | 2017 | 0 | 0 | 0 | 0 | 0 | - |
|  | 2018 | 0 | 0 | 0 | 0 | 0 | - |
|  | 2019 | 0 | 0 | 0 | 0 | 0 | - |
| **Netherlands** | 2017 | 13 | 313 | 0.25 | 0.09 | 0.66 | fear |
|  | 2018 | 53 | 18297 | 0.18 | 0.16 | 0.66 | fear |
|  | 2019 | 247 | 50095 | 0.17 | 0.15 | 0.68 | fear |
| **Poland** | 2017 | 25 | 6218 | 0.29 | 0.2 | 0.51 | trust |
|  | 2018 | 128 | 37125 | 0.18 | 0.17 | 0.65 | fear |
|  | 2019 | 63 | 3315 | 0.15 | 0.16 | 0.68 | fear |
| **Portugal** | 2017 | 196 | 83956 | 0.17 | 0.13 | 0.7 | trust |
|  | 2018 | 598 | 56779 | 0.18 | 0.13 | 0.69 | trust |
|  | 2019 | 170 | 60077 | 0.16 | 0.11 | 0.73 | fear |
| **Romania** | 2017 | 281 | 27585 | 0.17 | 0.16 | 0.67 | trust |
|  | 2018 | 164 | 13858 | 0.16 | 0.18 | 0.66 | fear |
|  | 2019 | 99 | 2920 | 0.14 | 0.19 | 0.67 | fear |
| **Slovakia** | 2017 | 5 | 479 | 0 | 0.08 | 0.91 | fear |
|  | 2018 | 9 | 1190 | 0.06 | 0.09 | 0.85 | trust |
|  | 2019 | 7 | 2631 | 0.11 | 0.19 | 0.7 | fear |
| **Slovenia** | 2017 | 7 | 1662 | 0.1 | 0.36 | 0.54 | fear |
|  | 2018 | 7 | 307 | 0.21 | 0.13 | 0.66 | trust |
|  | 2019 | 11 | 481 | 0.31 | 0.08 | 0.61 | fear |
| **Spain** | 2017 | 53 | 545272 | 0.26 | 0.15 | 0.59 | fear |
|  | 2018 | 165 | 114902 | 0.16 | 0.17 | 0.67 | fear |
|  | 2019 | 446 | 294439 | 0.17 | 0.16 | 0.67 | trust |
| **Sweden** | 2017 | 77 | 44532 | 0.2 | 0.09 | 0.71 | fear |
|  | 2018 | 119 | 18142 | 0.15 | 0.13 | 0.72 | fear |
|  | 2019 | 57 | 24284 | 0.17 | 0.23 | 0.6 | fear |
| **United Kingdom** | 2017 | 74 | 78426 | 0.21 | 0.18 | 0.6 | fear |
|  | 2018 | 155 | 359216 | 0.21 | 0.12 | 0.67 | fear |
|  | 2019 | 450 | 1574476 | 0.22 | 0.16 | 0.62 | fear |

Table S2
Values of coherence and perplexity for certain number of topics in the latent Dirichlet allocation model

| **number of topics** | **coherence** | **perplexity** |
| --- | --- | --- |
| **1** | 0.03231589 | 26963.736 |
| **2** | 0.03073071 | 22032.961 |
| **3** | 0.03710312 | 19022.549 |
| **4** | 0.03733930 | 17097.805 |
| **5** | 0.03443310 | 15608.449 |
| **6** | 0.03922612 | 14499.887 |
| **7** | 0.03357108 | 13670.281 |
| **8** | 0.03549733 | 13083.410 |
| **9** | 0.03961535 | 12419.925 |
| **10** | 0.03669200 | 11880.328 |
| **11** | 0.03326907 | 11437.530 |
| **12** | 0.03890998 | 11267.533 |
| **13** | 0.04589705 | 10793.677 |
| **14** | 0.04201717 | 10574.538 |
| **15** | 0.04111192 | 10458.178 |
| **16** | 0.04780655 | 10039.018 |
| **17** | 0.05107478 | 9900.571 |
| **18** | 0.04394814 | 9661.700 |
| **19** | 0.05664597 | 9371.237 |
| **20** | 0.05209300 | 9339.325 |
| **21** | 0.05669532 | 9141.601 |
| **22** | 0.05346645 | 9104.250 |
| **23** | 0.04337596 | 9011.315 |
| **24** | 0.05865064 | 8907.894 |
| **25** | 0.04919344 | 8836.616 |
| **26** | 0.05440027 | 8726.770 |
| **27** | 0.05375043 | 8522.443 |
| **28** | 0.05448442 | 8461.723 |
| **29** | 0.05234599 | 8503.750 |
| **30** | 0.05983814 | 8422.798 |
| **31** | 0.05579015 | 8339.439 |
| **32** | 0.05713496 | 8284.181 |
| **33** | 0.05790903 | 8175.807 |
| **34** | 0.05869199 | 8142.428 |
| **35** | 0.06221337 | 8181.046 |
| **36** | 0.05207640 | 8019.915 |
| **37** | 0.05694878 | 7978.959 |
| **38** | 0.05246757 | 8003.718 |
| **39** | 0.06853142 | 7925.832 |
| **40** | 0.06217627 | 7852.402 |

Table S3
Variance inflation factor (VIF) scores of the regression variables

| **variable** | **VIF score** |
| --- | --- |
| topic 1 | 1.031105 |
| topic 2 | 1.103315 |
| topic 4 | 1.061390 |
| topic 5 | 1.033456 |
| topic 6 | 1.053212 |
| topic 7 | 1.117317 |
| topic 10 | 1.133876 |
| topic 12 | 1.124203 |
| topic 13 | 1.060389 |
| anger | 3.703508 |
| joy | 2.578992 |
| surprise | 2.932670 |
| sadness | 4.910995 |
| disgust | 3.362078 |
| population | 1.297514 |
| social media users | 1.265097 |

Table S1
Measles-vaccine second-dose immunization coverage (WHO data)

| **Country** | **2017** | **2018** | **2019** |
| --- | --- | --- | --- |
| **Austria** | 84% | 84% | 84% |
| **Belgium** | 85% | 85% | 85% |
| **Bulgaria** | 92% | 87% | 87% |
| **Croatia** | 95% | 95% | 95% |
| **Republic of Cyprus** | 88% | 88% | 88% |
| **Czech Republic** | 90% | 84% | 84% |
| **Denmark** | 88% | 90% | 90% |
| **Estonia** | 91% | 88% | 90% |
| **Finland** | 92% | 93% | 93% |
| **France** | 80% | 83% | 83% |
| **Germany** | 93% | 93% | 93% |
| **Greece** | 83% | 83% | 83% |
| **Hungary** | 99% | 99% | 99% |
| **Ireland** | N/A | N/A | N/A |
| **Italy** | 86% | 89% | 88% |
| **Latvia** | 89% | 94% | 96% |
| **Lithuania** | 92% | 92% | 93% |
| **Luxembourg** | 86% | 90% | 90% |
| **Malta** | 83% | 95% | 95% |
| **Netherlands** | 90% | 89% | 90% |
| **Poland** | 93% | 92% | 92% |
| **Portugal** | 95% | 96% | 96% |
| **Romania** | 75% | 81% | 76% |
| **Slovakia** | 97% | 97% | 98% |
| **Slovenia** | 94% | 94% | 94% |
| **Spain** | 93% | 94% | 94% |
| **Sweden** | 95% | 95% | 95% |
| **United Kingdom** | 88% | 88% | 87% |

Table S2
Sample fragments of translated articles with a high intensity of the emotions

| **Emotion** | **Example** |
| --- | --- |
| **Fear** | No, measles is not a disease eradicated. The infection was a peak of recurrence. Attention danger! A viral infection not benign measles is classified among the diseases of childhood. It can be very dangerous in the fragile. Fragile persons have a high risk of complications. Pneumonia, neurological disorders, kératites, encephalitis. |
| **Trust** | The Management of the Health of the Wood, IASAUDE has confirmed two cases of measles in the Wood, advancing to the regional press. Note that on the last Sunday of the IASAUDE was notified of two potential cases of measles in the Wood, which at the time were under investigation and awaiting confirmation in the laboratory at the National Institute of Health Dr. Ricardo Jorge. The office of health it was noted that if it were two people of the female gender who are admitted to the Health Service of the Madeira “is a clinical condition is stable”, adding that “the Regional Health Authority, in co-ordination with the Directorate-General of Health and the National Institute of Health Dr. Ricardo Jorge, you are to keep up with the evolution of the situation. |
| **Anger** | Boris Johnson will today demand social media sites take down measles jab scare stories. The PM’s call comes as he launches a drive to boost vaccination rates — falling in the face of online claims. Boris Johnson will demand social media chiefs take down measles jab scare stories. He will announce he is hauling in tech bosses to a summit to order them to do more to end fake news about immunisation risks. He declared: “One case of this horrible disease is too many. I am determined to step up our efforts to tackle its spread.” Measles is rising again as child vaccinations drop. |
| **Sadness** | It was an adult between the ages of 30 and 40 years, informed the district of Hildesheim. In April, the Person had died from the effects of measles. All of the symptoms and the typical clinical picture indicated the Virus as the cause of death. A week earlier, a family member falls sick of the measles. Then, the deceased had taken the precaution of vaccination. The vaccination, however, came too late. Why the disease, however, led within days to the deaths, is now being examined carefully. |
| **Disgust** | The symptoms of measles appear 7 to 18 days after contact with an affected person. Measles is like a cold (cough, fever higher than 38°C, runny nose, red and watery eyes), and then appear red pimples on the face and on the rest of the body. |
| **Anticipation** | There is a need to be prepared for a vaccination campaign to identify the children who are not yet immunized against the illness, which will begin at the end of the week, and continued for ten days. In addition to this campaign, he continued, is already in place in the second week of April and the intensification of vaccination in the districts of Saurimo, capital of Lunda Sul and other municipalities in the province. "At the end of the week, this is what we're going to do an intervention on the big one: We have to do with the formation of the team to go all the way to the street. We expect a minimum of 100 teams, each team has four elements, two technicians, a support for the rally and one for track your kids vaccinated," he said. |
| **Surprise** | Despite the magnitude of the epidemic, there is an alarming lack of players and funds to respond to the measles outbreak underway in the DRC. With only $ 2.5 million mobilized on the $ 8.9 million needed to fund the humanitarian response, the contrast is striking with the outbreak of Ebola in the eastern part of the country which attracts many organizations and hundreds of millions of dollars. "So that rapid response and appropriate is crucial to contain the epidemic, particularly in children, there is mainly the lack of players on the field and a lack of support" warns Karel Janssens, head of mission MSF in the DRC. |
| **Joy** | The minister of Health has always been at the ceremony of awarding the Prize to Dr. University Carol of the year award was initiated in 2011 by the former minister of Health, Cseke Attila for the appreciation of the nurses, who are best known for our dedication and professionalism in their work. In this context, to Each their Own to launch a campaign for vaccination against measles, the campaign will probably take place from the 1st of June, the Birthday of the Child. |

Table S6
Examples of translated articles with a high proportion of the topic

| **Topic** | **Group classification** | **Example** |
| --- | --- | --- |
| **topic 1** | Education | The measles virus affect 'memory cells' of the immune system. As a result, patients sometimes several years after the infection was still in a lot of more comprehensible for other diseases. That is according to new Dutch research that is available to the RTL News report. The researchers of the Amsterdam UNIVERSITY medical center and the Wellcome Sanger Instute, Cambridge, came to that conclusion. They used blood samples from 77 children who had not been vaccinated, and the virus got in. The results, published Friday in the scientific journal Science, reveals that the measles virus in 11 to 73% of all antibodies fades away. The measles virus, which has so great an influence on the immune system. The researchers found that, especially, memory cells are erased. Which cells in the body to have a yeast infection and used later, to create a new infection to deal with. Due to the measles virus, many of these cells 'know', the body has the diseases are less of a good fight. |
| **topic 2** | World | At least 53 people, including 48 children under the age of four, have died in Samoa in the South Pacific, because of an outbreak of measles despite the efforts of the authorities to prevent the spread of this infectious disease. A total of 3.728 people have contracted this disease in Samoa from the start of the outbreak in October, 198 of them in the last 24 hours, the Government said samoan on Twitter. Latest update: 3,728 measles cases have been reported since the outbreak with 198 recorded in the last 24 hours. To date, 53 measles related deaths have been recorded. Since the Mass Vaccination Campaign on 20 Nov 2019, the Ministry has successfully vaccinated 58,150 individuals. |
| **topic 3** | World | New York, measles, mayor proclaims health emergency. Mayor De Blasio has stated that unvaccinated persons, of Brooklyn, will do so as soon as possible, under penalty of a fine of one thousand dollars From the month of September, more than 250 people in New York have contracted the measles, generating alarm, especially among the community of jewish orthodox Williamsburg. As set forth by mayor de Blasio, all unvaccinated persons in the four areas of Brooklyn should do it as soon as possible, under penalty of a fine of one thousand dollars. De Blasio has also ordered all religious schools and nurseries in the community, the jewish orthodox not to accept pupils and students are not vaccinated. After the epidemic, meanwhile, has been declared state of health emergency throughout the city. |
| **topic 4** | Education | Measles can result in serious complications but, thanks to vaccination programs, the incidence of outbreaks is low. Initial symptoms of measles can include: A runny or blocked nose Sneezing Watery eyes Swollen eyelids Sore, red eyes that may be sensitive to light A fever Small greyish white spots in the mouth Aches and pains Cough Loss of appetite Tiredness, irritability and a general lack of energy Contact your GP as soon as possible if you suspect that you or your child has measles. It’s also advised that you phone the surgery ahead of your visit, as they may need to make some arrangements to reduce the risk of spreading the infection to others. How do I Treat Measles? There’s no specific treatment for measles, but the condition usually improves within seven to 10 days. If the symptoms of measles are causing discomfort for you or your child, there are some things you can do to treat these while you wait for your body to fight off the virus. Rest and drink plenty of fluids to avoid dehydration. Paracetamol or ibuprofen can be used to reduce a fever and relieve any aches or pains if you or your child is uncomfortable. (Aspirin should not be given to children under 16). |
| **topic 5** | Education | ANDREW WAKEFIELD has a lot to answer for. He’s the man who in 1998 claimed — wrongly — that there was a possible link between the MMR vaccine and autism. His work has since been discredited and he has been struck off as a UK doctor. But in the meantime, take-up of the vaccine was massively affected — even though subsequent studies found NO link to autism. Andrew Wakefield spread lies about a link between the MMR vaccine and autism. Even Donald Trump seems to have wised up. In 2015 he ridiculously suggested that vaccines were responsible for what he called an “epidemic” of autism. Now he is telling Americans to “get their shots” because “vaccinations are so important”. This comes on the back of news that more people in the US have been infected with the measles in 2019 than any year since the disease was considered eradicated. |
| **topic 6** | Europe | Boris Johnson reacted after the number of cases of people with measles jumped. Boris Johnson has ordered urgent action to boost the number of children and young people receiving vaccinations following a rise in cases of measles. The Prime Minister will set out the plans to improve vaccination rates – including for the measles, mumps and rubella jab (MMR) – on a visit to a hospital in the South West on Monday. He has called for health leaders to renew their efforts to ensure 95% of the population have had both doses of the MMR vaccine. Currently only 87.2% of children have the second dose of the jab, down from a high of 88.6% in 2014-15, the lower uptake of which is thought to be partly behind the spread of measles, Downing Street said. There were 231 confirmed cases of measles in the UK during the first quarter of 2019, and Britain has lost its ‘measles-free’ status with the World Health Organization (WHO) three years after the virus was eliminated in the country. Advertisement Advertisement NHS England will write to all GPs urging them to promote ‘catch-up’ vaccination programs, and will seek to strengthen the role of local immunisation co-ordinators in a bid to improve uptake. The Government will also seek to update the advice on the NHS’s website to address misleading information about the dangers of vaccines. The UK lost its ‘measles-free’ status three years after the virus was eliminated in the country. |
| **topic 7** | Europe | In Brandenburg, the children in day-care centers and in day to care of mandatory measles vaccination. The vaccination could also be used for other infectious diseases is necessary. Who sends his child to a Brandenburg Kita, it is prior to mandatory measles vaccination. The want of the SPD, the Left, the CDU and requested the state government to look into this. Once this is done and approved, must be in the state of Brandenburg children in day-care centers and in day care up to a nationwide scheme against measles should be vaccinated. "The measles are still one of the most dangerous childhood diseases", - stated in the yesterday adopted a resolution. Measles are highly contagious and can lead to potentially fatal brain inflammation. Brandenburg wants to check if there are any mandatory vaccinations are also available against other infectious diseases is necessary. Least of all the SPD Federal Minister for family Affairs, Franziska Giffey, and the CDU had spoken-the Federal Minister of health, Jens Spahn for a measles vaccination. |
| **topic 8** | Europe | According to the information available, there were up to 19 hours of Wednesday morning, seven of those with the disease, and, in the course of the outbreak have been confirmed in 97 cases, most of them with a connection to the Santo António Hospital, in Porto. The DGS is that they are held to two patients to be clinically stable and that there are 20 cases being investigated. On Wednesday morning, the balance of the DGS pointed to the 90 confirmed cases of measles, on the current outbreak. “It is in the course of the investigation, the epidemiological details of a situation, which includes laboratory investigation of all cases,” says the organization, adding that to be on the keep track of, and in conjunction with the network of the ministers of health, the National Institute of Health Dr. Ricardo Jorge, and health care professionals, in the development of the situation in accordance with the provisions of the Contingency Plan. |
| **topic 9** | Europe | In the Italian parliament, in Rome, after an outbreak of measles in the country, the controversial mandatory vaccination for all children and young people under the age of 17 years, on Friday, approved. That was, however, a clear majority, with 296 votes against 92 votes against. Parents should check their children for ten diseases to be vaccinated next to measles, mumps, among others, against mumps, rubella (MMR), or rubella, whooping cough, and chickenpox. If the parents refuse, their children no longer go to the nursery and they can get a fine of 500 to 1000 euro to go up. There is an exception for children with a special medical history. The measles epidemy it has been since the beginning of the year in Italy, three killed-made. According to the official figures, to the middle of July 3.672 people have been infected. Only 87% of Italians are against measles vaccination. |
| **topic 10** | Europe | Europe is losing ground in the battle to eradicate measles. After several years of “steady progress” toward the elimination of the virus in the european region, “the number of Countries that have reached elimination of the disease is diminished. The four Countries in fact have lost the Status of a ‘measles-free’. This is the conclusion of the european Commission of verification of regional elimination of measles and rubella (RVC). The Commission's analysis is based on the data of the 2018 related to the 53 member States of the european Region. According to the results, the 4 Countries that have lost the status of a measles-free, or ‘free from measles’: Albania, Czech Republic, Greece and the United Kingdom. To certify the “dramatic recovery” of the movement are the recent data of the Who, disclosed two weeks ago: the number of cases of measles in Europe from January to June 2019, was about 90,000, double what it reported for the same period in 2018, and half occurred in the Ukraine, followed by Kazakhstan and Georgia. In 2017, approximately 110,000 people died of measles worldwide, mostly children under the age of 5 years. |
| **topic 11** | Europe | An 18-year-old died from measles, in Thessaloniki, greece. This is the fourth fatal case of the disease in our country. The 18-year-old afflicted by, though he was fully vaccinated and resulted from respiratory failure in the Intensive care Unit (ICU) of the hospital Papageorgiou. From complications of the disease have come up with a infant, a 17-year-old Roma and a 35-year-old mother from Attica. |
| **topic 12** | Europe | Measles has been a third death in France in 2018, a young girl of 17 years died "of the suites of neurological complications" related to this contagious disease to the university hospital of Bordeaux, announced Friday the regional Agency of health (ARS), New Aquitaine. "This is the 2nd death caused by this disease in New-Aquitaine and the 3rd in France since November 2017," says the ARS in a press release. And on the 23rd death due to the disease since 2008. Measles causes a 2nd death in New-Aquitaine. Two other deaths since the end of June the End of June, the public Health agency of France had announced the deaths due to measles in a patient of 26 years. The disease had already killed in February in Poitiers, a mother of 32 years who had never been vaccinated. According to the daily, the New Republic, she had contracted in the hospital, where she had led his father to a hospital. "The New-Aquitaine is no longer in phase of the epidemic" but the fight for the vaccination continues, insists the ARS. Since the November 6, 2017, 1096 cases of measles have been confirmed in the region (2567 in France in total). One out of four cases required hospitalization, twelve patients were transferred in intensive care and two died. |
| **topic 13** | Europe | Rome - On vaccines, the government takes a step back. The clamor of the critics collected in the announcement of the amendment to the draft on vaccines, which would delete the requirement to submit certifications vaccine at the time of enrollment in kindergartens and nursery schools, has done to change the idea to a coalition of yellow-green. "We think a modification of the amendment maintaining the mandatory certification of immunization for school attendance only for the measles," explains Pierpaolo Sileri (M5S), chairman of the health Commission of the Senate, where is the question of the ddl. The meeting point is therefore a step back for the measles, which, as underlined in the Sileri, "poses the biggest problems at this time." Besides, he adds, this vaccine is trivalent and covers well as from rubella and mumps". |
